# Mitigation of serotonin-caused suppression of osteoblast function by extracts of the psychoactive indigenous plant *Sceletium tortuosum*

**DOI:** 10.1186/s12906-026-05540-x

**Published:** 2026-08-22

**Authors:** Heinz C. Schröder, Matthias Wiens, Shunfeng Wang, Meik Neufurth, Stefan Louw, Oluwagbenga Johnson, Susanne Hoff, Xiaohong Wang, Werner E. G. Müller, Petrina Kapewangolo, Fransina Shafombabi

**Affiliations:** 1 ERC Advanced Investigator Group, Institute for Physiological Chemistry, University Medical Center of the Johannes Gutenberg University, Duesbergweg 6, Mainz, 55128 Germany; 2 NanotecMARIN GmbH, Mühlstr. 19, Ingelheim am Rhein, D-55218 Germany; 3 Department of Physics, Chemistry and Material Science, University of Namibia, Private Bag 13301, 340 Mandume Ndemufayo Ave, Pionierspark, Windhoek, Namibia; 4 Department of Mining and Metallurgical Engineering, University of Namibia, P.O. Box 3624, Eng. José Eduardo dos Santos Campus, Ongwediva, Namibia; 5 Department of Chemical Engineering, University of South Africa, Pretoria, South Africa; 6 Mediplants Namibia Cc, P.O. Box 2017, Windhoek, Namibia; 7 Department of Chemistry and Biochemistry, University of Namibia, Windhoek, Namibia

**Keywords:** Dietary supplements, Mineralization, Alkaline phosphatase, Serotonin, Serotonin reuptake inhibitor, Radical scavenging activity, SaOS-2 cells, Mesembrine-type alkaloids, *Sceletium tortuosum*

## Abstract

**Background:**

*Sceletium tortuosum* is an endemic plant in Southern Africa, traditionally used as kanna by the indigenous people and also exploited commercially due to its mood-elevating properties. Mesembrine, a major alkaloid from this plant, acts as a selective serotonin reuptake inhibitor (SSRI), a group of drugs commonly used in antidepressant therapy but with the undesirable side effect of impairing bone mineral formation. The aim of this study was to investigate the potential effects and mechanisms of extracts from this plant on mineralization.

**Methods:**

Both aqueous and ethanolic extracts were prepared from *S. tortuosum*. Their effects on mineralization were studied using bone-forming SaOS-2 cells. In addition to the effects on cell viability and alkaline phosphatase (ALP) activity, the radical scavenging activity of the extracts was determined using the 2,2-diphenyl-1-picrylhydrazyl (DPPH) and 2’,7’-dichlorodihydrofluorescein diacetate (H_2_DCF-DA) assays. The alkaloid composition was analyzed by liquid chromatography–mass spectrometry (LC-MS).

**Results:**

It is shown that the *S. tortuosum* extracts, in contrast to SSRIs, have a significant mineralization-promoting activity in vitro. This result was obtained despite the fact that, as is characteristic for SSRIs, the transient attenuation of the serotonin-induced suppression of cell viability that occurs with increasing serotonin concentrations was inhibited in SaOS-2. The activity of ALP, a marker of differentiated osteoblasts, remained unchanged. It was found that the plant extracts exhibit a pronounced radical scavenging activity not only in the DPPH assay, but also intracellularly using the cell-permeant probe H_2_DCF-DA. The alkaloid fraction of the aqueous extract of *S. tortuosum* consists primarily of the two bioactive alkaloids mesembrine and mesembrenone. Mesembrine proved to be only a weak inducer of mineralization.

**Conclusions:**

The results suggest that the observed osteogenic effects of *S. tortuosum* are due to a tuned interaction with signaling pathways involving serotonin receptors, serotonin transporters and reactive oxygen species. Due to its mineralization-promoting properties, we conclude that *S. tortuosum* is of potential interest for the long-term treatment of depressive disorders as an adjunct to antidepressant drugs that increase the risk of osteoporosis.

**Supplementary Information:**

The online version contains supplementary material available at 10.1186/s12906-026-05540-x.

## Background

Selective serotonin reuptake inhibitors (SSRIs) are widely used as antidepressants [1]. These drugs suppress the reuptake of serotonin (5-hydroxytryptamine, 5-HT) and thus increase the extracellular serotonin concentration by inhibiting the 5-HT transporter. It has been shown that long-term treatment of depression with SSRIs is associated with an increased risk of osteoporosis and bone fractures due to a reduction of bone mineral density even at therapeutic doses [2]. The 5-HT transporter is expressed both in osteoblasts, osteocytes and osteoclasts [3, 4] and is also functionally active in bone [3, 5, 6]. Therefore, it is of interest to investigate whether these adverse effects on osteoblast differentiation, proliferation and function/mineralization are also shown by medical plants/plant extracts showing antidepressant activity. A prominent member of this group of plants is *Sceletium tortuosum*, a succulent herb endemic to Southern Africa [7].

*S. tortuosum* (L.) N.E.Br., belonging to the family Aizoaceae (sub-family Mesembryanthemaceae), is tradionally used by the indigenous San (Bushmen) and Khoi people living in arid regions of Namibia, Botswana and South Africa due to its hunger and thirst relieving as well as mood-enhancing effects. It is also used for treatment of pain such as stomachache or toothache [8]. Based on experimental studies, various pharmacological properties of *S. tortuosum* extracts have been described including antidepressant, anxiolytic, analgesic, antiepileptic, and cognition improving activities [8, 9]. In in vitro experiments, *S. tortuosum* also shows a significant anti-HIV-1 (human immunodeficiency virus 1) activity [10]. Traditionally, this plant, also refered to as kanna, channa or kougoed [11], is mostly consumed by chewing but it can also be taken up by sniffing/smoking or as a tincture [9]. Commercially, *S. tortuosum* or its fermentation product is distributed as a powder or in the form of capsules named “kanna” for the treatment of depression and anxiety [12, 13].

The neurological activities of *S. tortuosum* are mainly caused by alkaloids present in the plant, the most well known being mesembrine [7]. This indole alkaloid [(3aS,7aS)-3a-(3,4-dimethoxyphenyl)-1-methyl-2,3,4,5,7,7a-hexahydroindol-6-one], whose structure has been previously elucidated [8, 14], acts as a potent 5-HT reuptake inhibitor [12, 15]. The toxicological safety of a standardized hydroethanolic extract of *S. tortuosum* has been assessed in pre-clinical and clinical studies [15–17]. Using this extract, no mortality or adverse effects have been observed in vivo, in rats, in a 14-day oral toxicity or 90-day subchronic oral toxicity study [17]. In a first clinical study on healthy volunteers, the same extract was found to be safe and well-tolerated over a 3-month period [16]. In a further double-blind, placebo-controlled study, a single administration of this extract in healthy individuals attenuated subcortical responsivity to threat; this supports the potential anxiolytic effect of the extract [15]. In addition, *S. tortuosum* extracts have been reported to possess free radical scavenging activity in DPPH (2,2-diphenyl-1-picrylhydrazyl) assay, as well as inhibitory activity against monoamine oxidase type B (MAO-B), acetylcholinesterase and *N*-methyl-d-aspartate (NMDA) receptor [18].

It is well known that, in addition to its function as a neurotransmitter, serotonin is involved in the regulation of a number of non-neuronal activities, in particular bone metabolism [19]. This biogenic amine, produced in the gut and released in the circulation, inhibits the proliferation and differentiation of osteoblasts and decreases bone mineral density [20]. Serotonin is also synthesized by bone cells [19]. It has also been shown to affect the proliferation and function of osteoblast-like SaOS-2 cells, used in the present study, even at concentrations in the physiological range; a significant suppression of SaOS-2 cell proliferation was detected at concentrations found in serum of patients receiving antidepressant therapy [21]. These effects are mediated by the presence of serotonin receptors on osteoblasts [19]. Therefore, serotonin has been implicated in the increased incidence of osteoporosis in patients receiving long-term treatment with SSRIs [22–25].

In a previous study, Kapewangolo et al. [10] has shown that *S. tortuosum* is rich of polyphenols, terpenes, tannins, alkaloids, anthraquinones, glycosides, carbohydrates anthocyanin, and coumarins, some of them potentially showing antioxidative activity. Oxidative stress caused by reactive oxygen species (ROS), such as superoxide radicals (O_2_^•−^), leads to the formation of hydrogen peroxide (H_2_O_2_) by superoxide dismutase (SOD); this—*via* Fenton reaction—triggers the intracellular generation of hydroxyl radicals (^•^OH), which are even more reactive. Therefore, it is important to know not only the extracellular radical scavenging activity exhibited by *S. tortuosum* extract [10] but also its intracellular antioxidative activity, especially because ROS are also involved in regulation of bone cell proliferation, differentiation and function [26].

In this study, we investigated the effect of *S. tortuosum* extracts on mineralization using bone-forming SaOS-2 cells as a model, examining this effect in relation to cell growth or viability as a function of serotonin concentration. Furthermore, the radical-scavenging activity of the extracts was analyzed—not only extracellularly *via* the DPPH assay but also in tests using the cell-permeable probe 2’,7’-dichlorodihydrofluorescein diacetate (H_2_DCF-DA). In some experiments, the plant extracts were encapsulated in a *N*,*O*-carboxymethyl chitosan (*N*,O-CMC) hydrogel matrix, which has previously been shown to enhance the stability of the active ingredients, e.g. to external, ultraviolet (UV) light stress (Schröder et al., to be published). Encapsulation into this matrix also intends to prevent degradation, e.g. during food processing, or gastric passage. Based on the results, a mechanism is proposed, explaining the unexpected, apparently contradictory effect of *S. tortuosum* extracts on bone forming SaOS-2 cells.

## Methods

### Materials

SaOS-2 cells (human osteogenic sarcoma cell line; #89050205), serotonin hydrochloride (#H9523), sertraline hydrochloride (#5.06188), fluoxetine (#TA9491596647), 2,2-diphenyl-1-picrylhydrazyl (DPPH; #D9132), *p*-nitrophenyl phosphate - disodium hexahydrate (#N9389), quercetin (#Q4951), β-glycerophosphate disodium salt hydrate (#G9422), l-ascorbic acid (#A4403), dexamethasone (#D1756), Alizarin Red S (#A5533), chitosan (from shrimp shells, ≥ 75% deacetylated; #C3646) and sodium alginate (alginic acid from the brown algae giant kelp *Macrocystis pyrifera*; #W201502) were obtained from Sigma-Aldrich (Taufkirchen; Germany). OsteoImage Mineralization Assay was from Lonza (Cologne, Germany). CaCl_2_·2H_2_O (#5239) was from Roth (Karlsruhe, Germany). PrestoBlue Cell Viability Reagent (#A13261) and heat-inactivated fetal calf serum (FCS; #A3840001) were from Thermo Fisher Scientific (Dreieich, Germany). Mesembrine [(3aS,7aS)-3a-(3,4-dimethoxyphenyl)octahydro-1-methyl-6 H-indol-6-one; purity > 98%] was purchased from US Biological (#282040, Salem, MA). A stock solution was prepared in ethanol (1 mg/mL) and diluted to the indicated concentrations in ethanol or cell culture medium/FCS.

### Plant material

The *S. tortuosum* powder prepared from the dried whole aerial parts of *S. tortuosum* was a gift from Mediplants Namibia Cc (Windhoek, Namibia), a company which commercializes capsules of medicinal plants produced by Farm Vredelus Pty Ltd (Mariental, Namibia). This farm is specialized in cultivation of desert plants from Namibia with medicinal properties. The identification, growth conditions, harvesting and processing (cleaning, drying and milling) have been described previously [10].

### Preparation of plant extracts

Both organic (ethanol) and aqueous extracts from *S. tortuosum* were prepared. The ethanol extract from *S. tortuosum* was prepared as described previously [10]. In brief, plant powder prepared from dried plant parts was agitated in ethanol at room temperature for 24 h. After filtration and concentration under vacuum, the extract was dried and stored at room temperature until use. The yield of extraction was 184.7 mg/g dry weight of plant material. To better simulate the in vivo chewing consumption, an aqueous extract was additionally prepared. The aqueous extract was obtained by macerating 10 g of *S. tortuosum* plant material in 200 mL of purified (Milli-Q) water for 24 h at room temperature with intermittent agitation. The mixture was filtered and the filtrate was dried with a freeze dryer for 24 h. The yield of the aqueous extraction was 313.2 mg/g dry weight of plant material.

For the experiments, the dried *S. tortuosum* extracts were dissolved in ethanol or cell culture medium (McCoy’s medium; #M4892, Sigma-Aldrich) supplemented with 10% FCS for 24 h at room temperature. In a further series of experiments, the dried plant extract (ethanol extract) was suspended in buffers of different pH (“acid buffer”: 0.1 M acetic acid pH 3.5; “neutral buffer”: McCoy’s cell culture medium pH 7.4; “basic buffer”: 0.1 M Na_2_HPO_4_ pH 9). After 24 h at room temperature and centrifugation (5,000 rpm; 5 min), the supernatants were sterile filtered using a sterilizing grade filter with a pore size of 0.2 μm.

### Preparation of *N*,*O*-carboxymethyl chitosan

The method for preparing the *N*,*O*-carboxymethyl chitosan (*N*,*O*-CMC) used for encapsulation of the plant extracts has been described previously [27]. In brief, chitosan was suspended in a water-isopropanol (1 : 2, v/v) mixture containing NaOH. Monochloroacetic acid dissolved in isopropanol was then dropwise added to the material. After heating, the mixture was filtrated, washed in ethyl alcohol, neutralized and dried. The product was sterilized by UV irradiation at 254 nm. The degree of substitution by carboxymethyl groups at the amino and primary hydroxyl moieties of chitosan in the *N*,*O*-CMC products was analyzed by Fourier transform infrared (FTIR) spectroscopy using an attenuated total reflectance-FTIR spectroscope/Varian IR spectrometer (Agilent, Santa Clara; CA) [27].

### Cells

Osteoblast-like human osteogenic sarcoma (SaOS-2) cells [28] were cultured in McCoy’s medium with 5% FCS in 25 cm^2^ flasks or 24-well cell culture plates (Thermo Fisher Scientific). Routinely, the cells were seeded at a density of 20 000 cells per 3 mL well and cultivated for 3–5 days in medium/FCS in a humidified incubator at 37 °C and 5% CO_2_.

### Cell viability assay

The effect of the *S. tortuosum* extracts on the growth/viability of SaOS-2 cells was determined using the fluorometric PrestoBlue assay as described [29]. The dried and pulverized extracts dissolved in medium/FCS and different dilutions in medium/FCS were prepared after sterile filtration as described above. A SaOS-2 cell suspension (20 000 cells/mL) was seeded into the wells of a 96-well black plate (200 µL/well). After cultivation for 3 d, the culture medium was replaced with medium containing the differently diluted plant extracts. Cell culture medium/10% FCS alone was used as control. After cultivation for 3 days, PrestoBlue reagent (20 µL/well) was added and incubation was continued for 30 min. The fluorescence caused by the conversion of resazurin to resorufin was measured at excitation 560 nm, emission 590 nm.

In order to study the effect of serotonin in the absence or presence of the plant extract (dried ethanol extract in medium/FCS at a final concentration of 30 µg/mL and 100 µg/mL) on growth/viability of SaOS-2, a stock solution of serotonin hydrochloride (10 mg/mL) in cell culture medium supplemented with 10% FCS was prepared and diluted with medium/FCS to 1 mg/mL, 100 µg/mL, 10 µg/mL, 1 µg/mL, 100 ng/mL and 10 ng/mL. A SaOS-2 cell suspension (20 000 cells/mL was added into the wells of a 96-well plate (180 µL/well). After overnight incubation at 37 °C, 20 µL of the differently diluted serotonin samples were added and cultivated for 48 h. At the end of the incubation period, the cell viability was checked with the PrestoBlue assay.

### Growth of SaOS‑2 cells onto hydrogel containing plant extract

*N*,*O*-CMC or Na-alginate was used as hydrogel. For encapsulation of the *S. tortuosum* extracts into *N*,*O*-CMC hydrogel, different concentrations of the extracts were mixed with *N*,*O*-CMC dissolved in cell culture medium at a final concentration of 50 mg/mL. Besides the ethanol extract in cell culture medium (“neutral buffer”), the dried extract treated with 0.1 M acetic acid pH 3.5 (“acid buffer”) or 0.1 M Na_2_HPO_4_ pH 9 (“basic buffer”) was used for encapsulation after centrifugation and sterile filtration. *N*,*O*-CMC solution alone was used as control. After placing the mixtures (20 µL) in the wells of a 96-well plate, the *N*,*O*-CMC was crosslinked by addition of 20 µL of 0.1 M CaCl_2_ solution. After incubation for 10 min and three times washing with distilled water, the hydrogels formed containing the plant extracts or control were overlayed with 180 µL/well of SaOS-2 cell suspension (10 000 cells/mL) in McCoy’s medium/5% FCS. Cultivation was for 3 days followed by the fluorometric PrestoBlue assay, as described above.

For encapsulation into alginate, a 2% (w/w) solution of sodium alginate in distilled water was prepared under stirring overnight. After mixing the extracts with the alginate solution, 100 µL aliquots of the mixtures were layered into the wells of a 24-well cell culture plate. Alginate alone was taken as control. Then crosslinking was performed for 30 min by addition of 500 µL of an aqueous 5% (w/w) CaCl_2_·2H_2_O solution. After washing twice with McCoy’s medium/10% FCS, the formed alginate hydrogels were overlaid with 100 µL of SaOS-2 cells in McCoy’s medium/10% FCS (40 000 cells/mL) and incubated overnight in a humidified incubator at 37 °C / 5% CO_2_ [27, 30]. Subsequently, the cells were stained with Calcein AM; after an incubation period for 3 days, they were stained again with the fluorescent dye.

### Extracellular radical scavenging activity

The DPPH scavenging assay was used to determine the extracellular free radical scavenging activity of the *S. tortuosum* extracts, essentially as described [31]. The DPPH solution dissolved in absolute ethanol (100 µg/mL) was added to increasing concentrations of ethanolic solutions of the dried *S. tortuosum* extracts at an 1:1 ratio. In addition, mesembrine (pure compound) dissolved in ethanol was tested. The radical scavenging activity of quercetin (in ethanol) was measured in parallel. After incubation for 30 min at room temperature with light protection, the absorbances at 517 nm were measured. The DPPH scavenging activity was calculated using the formula

$$\mathrm{Inhibition}\;\left(\%\right)=\left(1-\frac{\mathrm{As}-\mathrm{Ab}}{\mathrm{Ac}-\mathrm{Ab}}\right)\cdot100$$

where As is the absorbance of the reaction with the sample, Ab the absorbance of blank, and Ac the absorbance of control (reaction with ethanol only). From these values, the IC_50_ values (50% reduction of free radical DPPH) were calculated.

### Intracellular antioxidant activity

The intracellular antioxidant activity of the extracts was determined using the Cellular Antioxidant Assay kit (ab242300; Abcam, Cambridge, UK) according to instructions of the manufacturer. The SaOS-2 cells (1 · 10^5^ cells/mL) were cultivated for 24 h in a 96-well black fluorescence cell culture plate (200 µL per well) until reaching 90% confluency. After washing with PBS, 50 µL of the H_2_DCF-DA working solution (10 µM; prepared by dilution of the H_2_DCF-DA stock solution with cell culture medium without FCS) was added to the cells, followed by addition of 50 µL of the plant extract or 50 µL of a quercetin standard series solution in cell culture medium. After an incubation period at 37 °C for 60 min and washing the cells three times with PBS, 100 µL of Free Radical Initiator working solution was added. The changes of the fluorescence intensity were monitored every five minutes over 1 h at 480 nm excitation and 530 nm emission. The data analysis was performed according to the Assay Kit manual. The Cellular Antioxidant Activity (CAA) units were calculated from the relative fluorescence value (RUF) of time point 0, 1, 2, …, 12, using the formula

$$\begin{aligned}\mathrm{CAA}=100&-\frac{\mathrm{AUC}\;\mathrm{antioxidant}}{\mathrm{AUC}\;\mathrm{control}}\cdot100,\\\mathrm{where}\;\mathrm{AUC}&=1+\frac{RUF1}{RUF0}+\frac{RUF2}{RUF0}\\&+\frac{RUF3}{RUF0}+...+\frac{RUF12}{RUF0}\end{aligned}$$

### Mineralization

Increasing concentrations of the *S. tortuosum* extracts (in cell culture medium/FCS) were mixed with *N*,O-CMC dissolved in cell culture medium and added into the wells of a 96-well microplate. After crosslinking with 0.1 M CaCl_2_ solution, 180 µL of SaOS-2 cell suspension (1 · 10^4^ cells/mL) was added onto the formed *N*,O-CMC hydrogel containing the plant extract. After cultivation of the cells for three days, medium/FCS supplemented with mineralization activation cocktail (MAC; containing 10 mM β-glycerophosphate, 0.2 mM l-ascorbic acid and 0.1 µM dexamethasone [32]) was added and incubation was continued for 5 days as outlined. The formation of hydroxyapatite crystallites was detected by staining with the “OsteoImage” dye as described previously [33] following the protocol of the manufacturer. The “OsteoImage” dye specifically binds to hydroxyapatite nodules deposited on the cells. Quantitative assessment of ossification was performed with the Alizarin Red S spectrophotometric assay by measuring the optical density at 405 nm [34, 35]. The values were normalized to total DNA determined with the PicoGreen method and calf thymus DNA as a standard [36].

### Alkaline phosphatase activity

SaOS-2 cells (100 000 cells/mL) were seeded into the wells of a 48-well plate (0.5 mL/well). After a pre-incubation period of 24 h, 0.5 mL of *S. tortuosum* extract (different concentrations) in medium/FCS with or without the MAC was added and incubation was continued for 3 or 5 days. Controls contained cell culture medium alone. Thereafter, the cells were washed three times with PBS and lysed with 0.5 ml ice-cold lysis buffer (10 mM Tris-HCl, 2 mM MgCl_2_ and 0.1% Triton-100, pH 10) by applying three freeze and thaw cycles. After centrifugation (13 000 x *g*, 10 min, 4 °C), the alkaline phosphatase (ALP) activity in the supernatants was measured colorimetrically at 405 nm (1:10 dilution) after addition of 100 µL of 5 mM *p*-nitrophenyl phosphate (*p*-NPP) and 100 µL of cell lysis buffer and incubation of the samples at room temperature for 1 h in the dark, as described [37].

### Liquid chromatography – tandem mass spectrometry

The dried *S. tortuosum* aqueous extract was re-dissolved in water (ca. 1.6 mg/mL) and filtered using a Millipore Millex-HV Hydrophilic PVDF 0.45 μm syringe filter (Merck, Germany). LC-MS and LC-MS^E^ analyses were performed on a Waters Acquity UPLC system consisting of a binary pump, degasser, auto-sampler and column oven, interfaced through an electron spray ionisation (ESI) source to a Waters Select Series Cyclic IMS quadrupole - time-of-flight (Q-TOF) high resolution mass spectrometer. LC-MS instrument control and data acquisition were performed using Waters’ MassLynx (version 4.2) software. All experiments were performed using a Waters BEH C_18_ column with dimensions of 100.0 mm length × 2.1 mm I.D. packed with 1.7 μm particles. An injection volume of 1 µL was used and the column and sample temperatures were maintained at 50 °C and ca. 25 °C (room temperature), respectively. The mobile phases used were *A*, 0.1% formic acid in 5% acetonitrile and *B*, 0.1% formic acid in pure acetonitrile, with a linear gradient of 0 (hold 0.5 min.) – 50% *B* in 11.5 min. A mobile phase flow rate of 0.35 mL/min was used. The samples were analysed in ESI positive mode, with a cone voltage of 15 V. Full scan MS data, where ions formed in the ion source are measured and no fragmentation occurs in the collision cell, were acquired with a scan range of 50–1500 amu and lock mass of 556.2766 amu. MS^E^ data (an acquisition mode performed concurrently with full scan acquisition in a single analysis, where fragmentation is performed using a collision energy ramp of 20–70 V to produce tandem mass spectra) was also acquired across a mass range of 50–1500 amu. Compounds were tentatively identified based on the comparison of their molecular formulae, calculated from the HR-MS data, to those reported in the literature for compounds previously identified in *S. tortuosum* [8, 38]. In addition, the MS^E^ fragmentation patterns were compared to those in databases such as COCONUT, KNApSAcK, Pubchem, Natural Products etc. using SIRIUS (Version 5.8.5), for additional evidence of the compounds’ identities [39].

### Total phenolic content

Total phenolic content was determined using the Folin-Ciocalteu method [40] as previously described [10]. The calibration curve was constructed using various dilutions of a gallic acid solution. Total phenolic content was expressed as gallic acid equivalents (GAE; in mg) per g of extract (mg GAE/g extract).

### Microscopic analyses

Light microscopical images were taken with a VHX-1000 Digital Microscope from Keyence (Neu-Isenburg; Germany).

### Statistical analysis

The statistical analysis was performed by using R-4.6.0 software [41]. The results were analyzed either by using the independent two-sample Student’s *t*-test after verifying that the values follow a standard normal Gaussian distribution or, in experiments involving multiple groups, multiple concentrations or repeated comparisons, by using one-way analysis of variance (ANOVA) or two-way ANOVA with Tukey’ test as a post-hoc test and the Holm correction, where indicated. The results are presented either as means ± standard deviation (SD) of at least three independent experiments or as the mean of three independent repeated experiments ± standard error of the mean (SEM). Values of *p* considered significant were expressed as *p < 0.05, **p < 0.01, and ***p < 0.001.

## Results

### UV spectrum profile of *S. tortuosum* extract

**Fig. 1:**
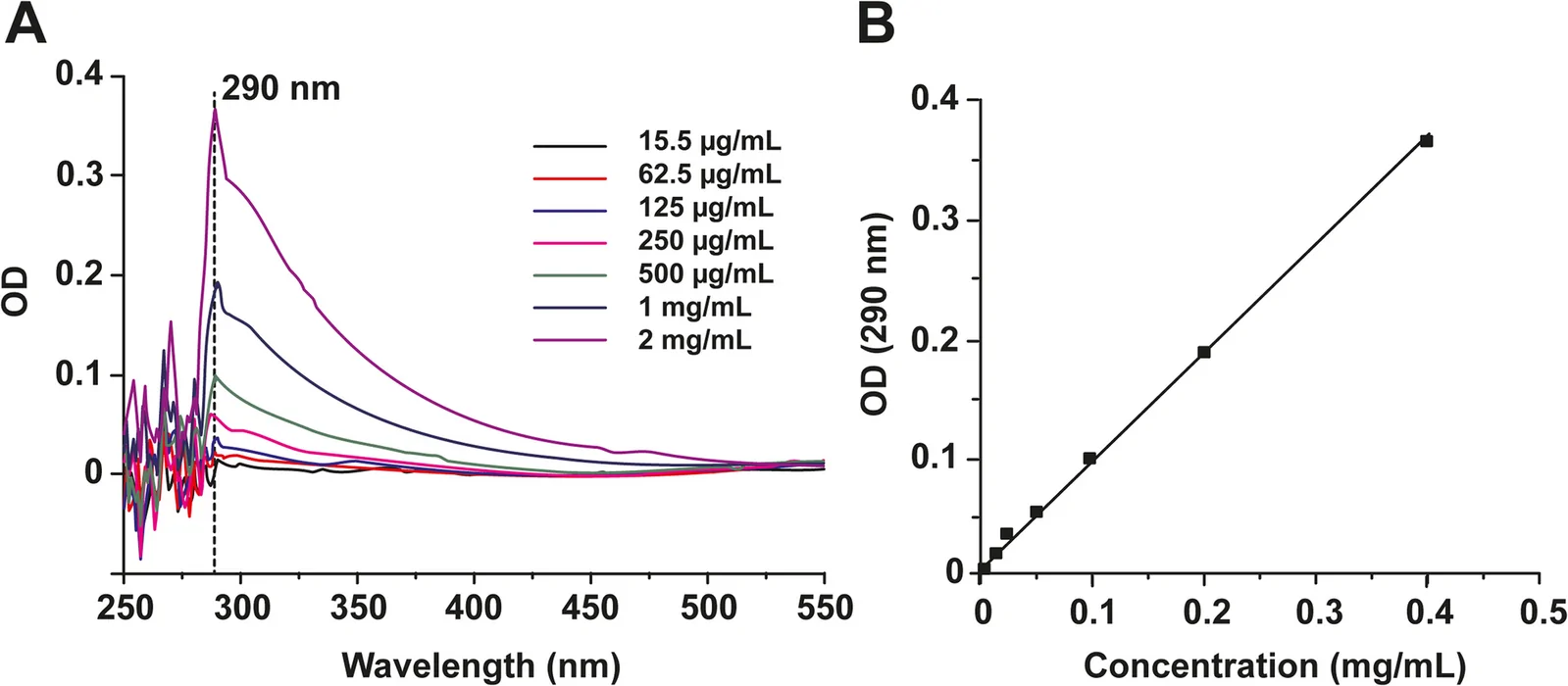
UV spectra of different dilutions of *S. tortuosum* extract in medium without FCS. **A **UV spectra in the wavelength range 250 to 550 nm. **B **Optical density of the plant extract plotted against the concentration of the extract

For the cell culture studies, the dried extracts of *S. tortuosum* dissolved in cell culture medium (McCoy’s medium)/FCS and sterile filtered before the experiments were used. The UV spectra of different concentrations of the ethanol extract dissolved in medium without FCS are shown in Fig. 1A. The optical density at 290 nm of the extract in cell culture medium linearly depended on the concentration of the plant material (Fig. 1B).

### Effect of the encapsulated *S. tortuosum* extracts on cell viability

**Fig. 2:**
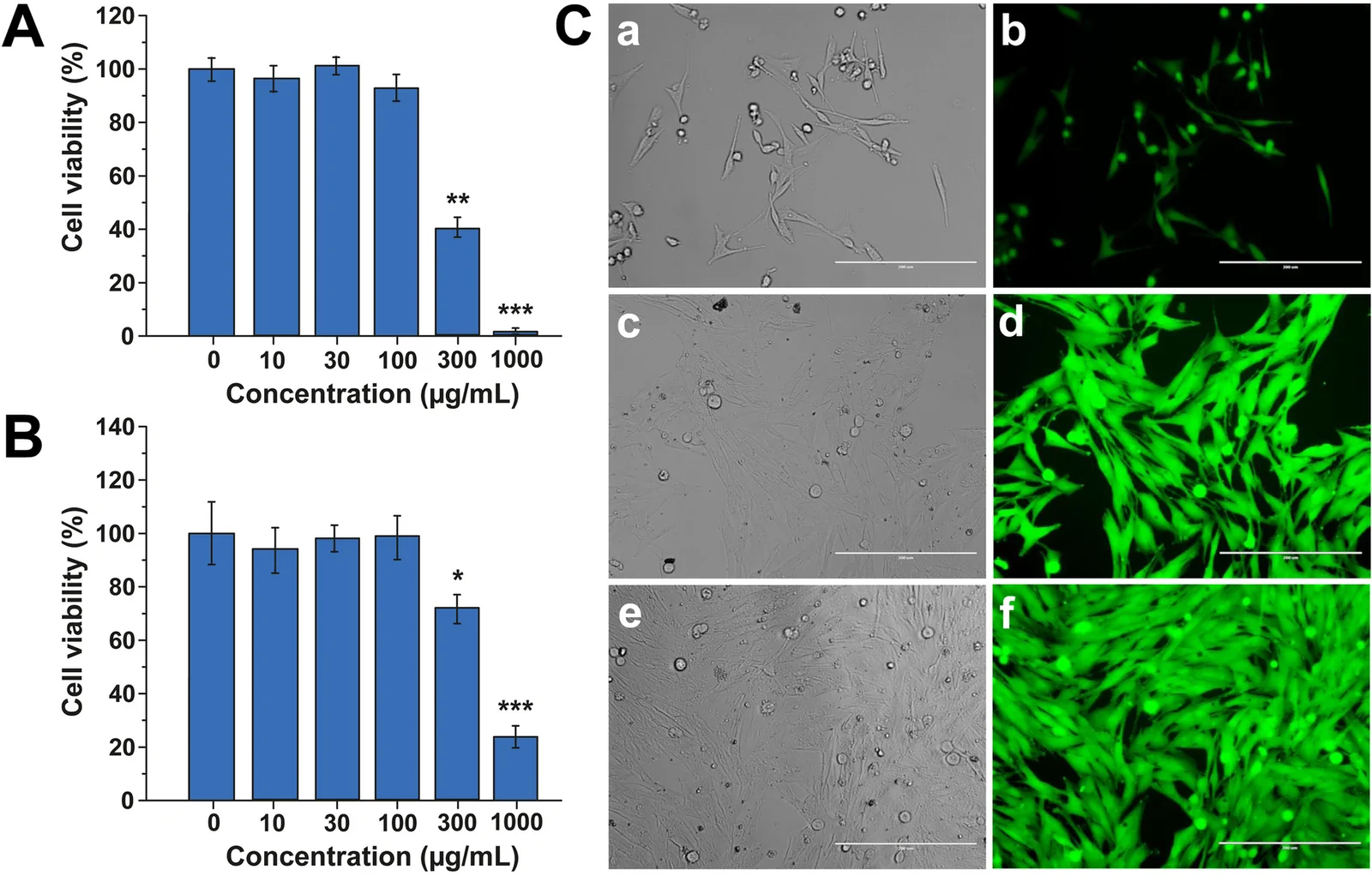
Growth/viability of SaOS-2 cells exposed to *S. tortuosum* extracts. **A **Effect of the *S. tortuosum* ethanol extract dissolved in cell culture medium supplemented with 10% FCS. **B **Effect of the aqueous extract in medium/FCS. Data are presented as the means ± SD (*n* = 10) of the samples with the respective extract concentrations compared to the sample without extract (control; cell culture medium alone); *p < 0.05, **p < 0.01, ***p < 0.001 (Student’s *t*-test). **C **Growth of SaOS-2 cells on alginate hydrogel supplemented without and with the dried *S. tortuosum* ethanol extract. Cells were visualized by live staining with Calcein AM (green fluorescence). (a and b) Images taken immediately after addition of the cells (gel without plant extract). (c to f) Cells grown on alginate hydrogel without (c and d) and with (e and f) extract after a 3 d incubation period. (a, c, e) Nomarski optics; (b, d, f) fluorescence microscopy images. Scale bars, 200 μm

The effects of the plant extracts were tested either directly or after incorporation into a hydrogel. In order to study the direct effect of the extracts on growth/viability of SaOS-2 cells, the dried ethanol and aqueous extracts were dissolved with medium/serum. The PrestoBlue assay was used, which measures the metabolic activity of the cells as an indicator of cell viability. As shown in Fig. 2A and B, exposure of the cells to both the ethanol and aqueous extracts of *S. tortuosum* up to a concentration of 100 µg/mL did not result in a significant decrease in the metabolic viability during the 3 d incubation period, compared to control without extract. At an extract concentration of 300 µg/mL, the reduction of cell viability was about 69.0% (ethanol extract) and 27.1% (aqueous extract), respectively. Addition of the ethanol extract at a concentration of 1000 µg/mL inhibited the cell growth/viability almost totally (by 98.4%); the inhibition by the aqueous extract was 76.0%.

In addition, the effect of the dried extracts embedded in an alginate hydrogel on growth of SaOS-2 cells was determined. The ethanol extract was mixed with the alginate solution and allocated into the wells of a 24 well plate as described in Methods. After hydrogel formation by addition of a Ca^2+^ solution, SaOS-2 cells (40 000 cells per well) were seeded onto the hydrogel and subjected to live staining with Calcein AM (Fig. 2C). At the beginning of the incubation period, only a weak staining of the (smaller) number of cells on the hydrogel is seen (Fig. 2C, a and b), while after cultivation of the cells for 3 d, both the cells grown on the hydrogel without plant extract (Fig. 2C, c and d) and the cells grown on the hydrogel with the incorporated plant extract (Fig. 2C, e and f) were intensively stained by the fluorescent dye, showing that the cells, at the concentration tested, remain alive during exposure to the plant material. Similar results were obtained with the aqueous extract (not shown).

### Effect of pH on cell viability

**Fig. 3:**
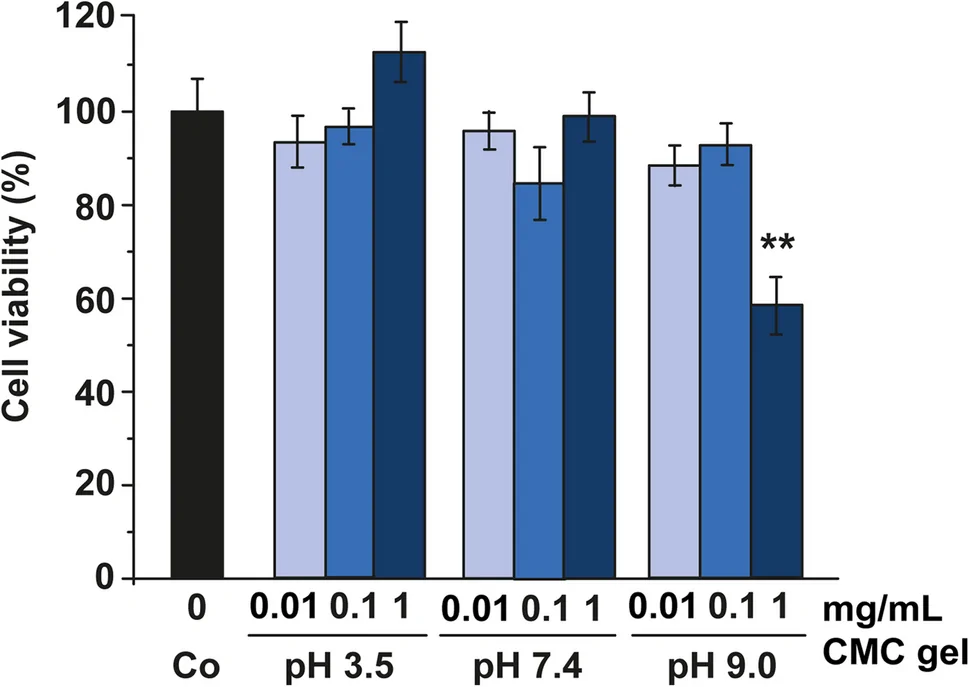
Dependence of viability of SaOS-2 grown onto the hardened *N*,*O*-CMC hydrogels containing different dilutions of *S. tortuosum* extracts prepared with buffers of different pH (pH 3.5, pH 7.4, and pH 9). The PrestoBlue assay was performed after an incubation period of 3 days. The results are expressed as % of the control (Co) without plant extract and are presented as the mean ± SD (*n* = 10). The significances of the data for the respective extract concentrations compared to the control (without extract) were calculated using Student’s *t*-test; **p < 0.005, compared to control

To examine the effect of the pH of the extraction medium on cell viability, the *S. tortuosum* extracts were incorporated into a *N*,*O*-CMC hydrogel matrix as described in Methods. The results showed that incubation of SaOS-2 cells for 3 days on the hydrogel matrix supplemented with increasing concentrations of the pH 3.5 and pH 7.4 extracts did not significantly affect the growth/viability of the cells, but rather had a slight, but not significant stimulatory effect at the highest concentration tested (pH 3.5 extract); Fig. 3. On the other hand, the hydrogel matrix containing the pH 9 extract with the lowest dilution level acted inhibitory on the growth/viability of the cells.

### Antioxidant activity of plant extract

**Fig. 4:**
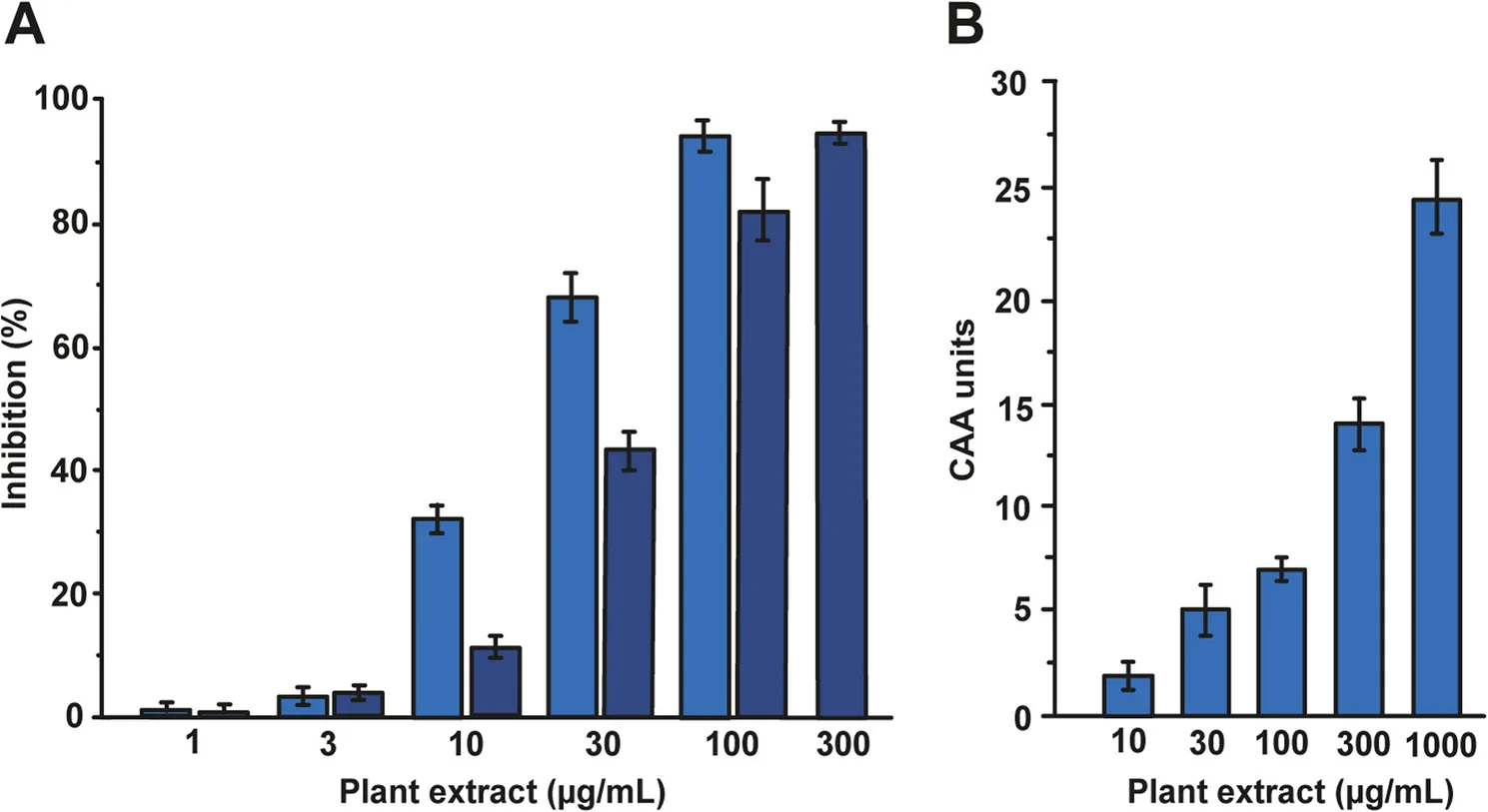
Antioxidant activity of *S. tortuosum* plant extracts. **A **DPPH free radical scavenging activity of the ethanol extract (light blue) and aqueous extract (dark blue). Values are means ± SD of eight replicate measurements each. **B **Intracellular antioxidant activity of dried *S. tortuosum* extract (ethanol extract dissolved in medium/FCS) using SaOS-2 cells. The results are presented in CAA units, which were calculated as described in Methods. Mean values ± SD of three independent experiments

Measurements of the antioxidant activity of the *S. tortuosum* extracts using the DPPH assay revealed a significant free radical scavenging activity (Fig. 4A). The IC_50_ value for the ethanol extract was reached at a concentration of the extract of 16.7 µg/mL. For the aqueous extract, an IC_50_ value of 50.8 µg/mL was obtained. Quercetin used as a reference material had an IC_50_ of 6.4 µg/mL (not shown).

In order to determine the intracellular antioxidant activity in SaOS-2 cells, the *S. tortuosum* ethanol extract in medium plus 10% FCS and the cell-permeant probe, 2’,7’-dichlorodihydrofluorescein diacetate (H_2_DCF-DA) were used. A quercetin standard solution was tested in parallel. In the presence of ROS, the H_2_DCF-DA probe is rapidly converted after deacetylation to a highly fluorescent compound. It was found that the *S. tortuosum* extract also shows a significant antioxidant activity in the H_2_DCF-DA assay (Fig. 4B). At a concentration of 1 mg/mL, the antioxidant activity of the extract amounted to 24.4 CAA units (Fig. 4B). A similar CAA value was reached by quercetin (pure compound) at a concentration of about 20 µg/mL (not shown). No DPPH scavenging activity was found for mesembrine (pure compound dissolved in ethanol; 10 µg/mL) (not shown).

### Effect of plant extract on mineralization of SaOS-2 cells

**Fig. 5:**
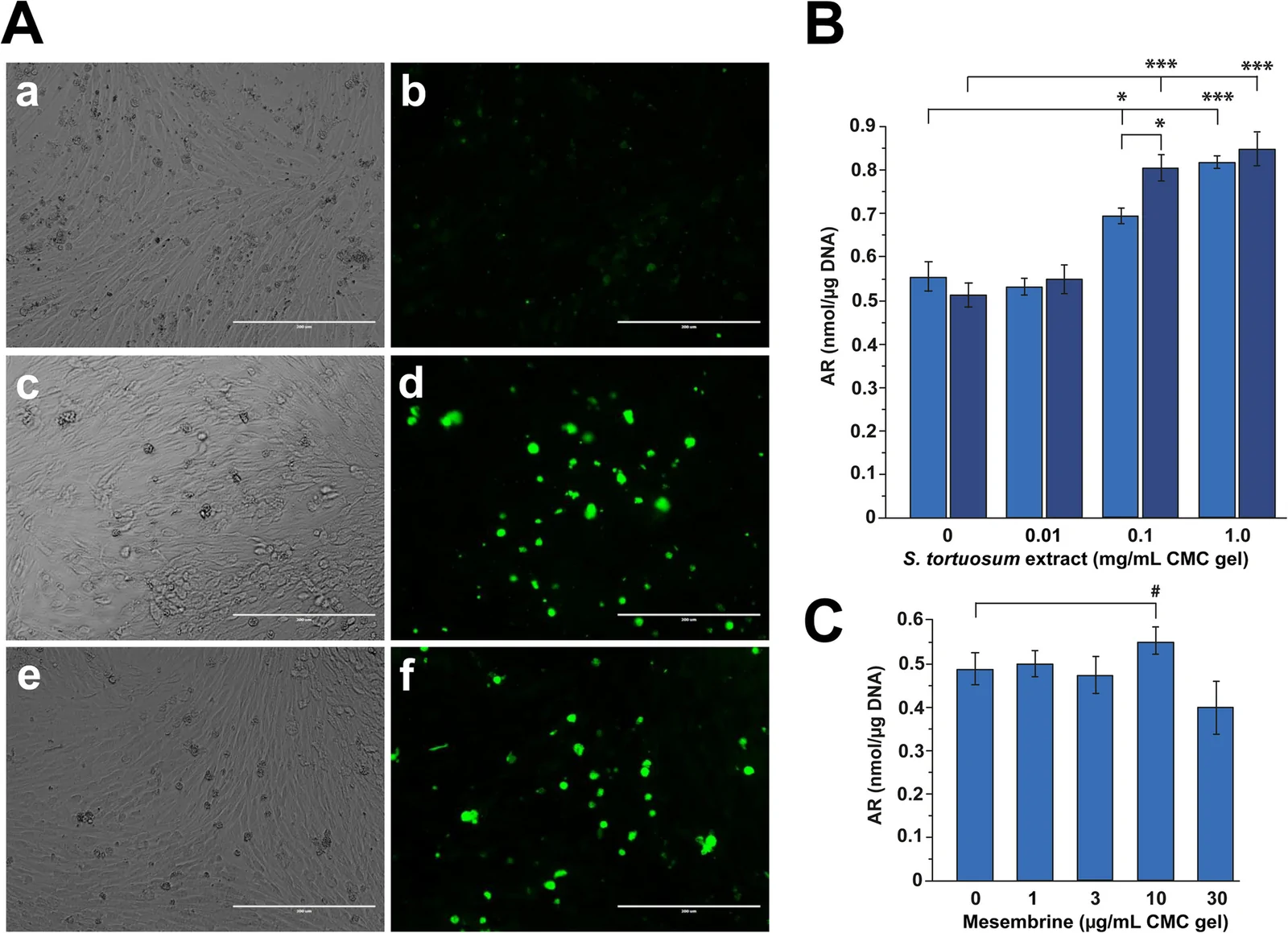
Mineralization of SaOS-2 cells grown onto *N*,*O*-CMC hydrogels supplemented with *S. tortuosum* extract. **A **Staining with “OsteoImage” dye. SaOS-2 grown onto *N*,*O*-CMC hydrogel supplemented with 0.1 mg/mL of the *S. tortuosum* ethanol extract were incubated in medium/FCS without (a and b) or with mineralization activation cocktail (MAC; c to f) in the absence (a to d) or presence of extract (e and f) for 5 days. The cells exposing hydroxyapatite nodules/crystals were then stained with “OsteoImage” dye. (a, c, e) Bright field images. (b, d, f) Corresponding fluorescent images. Scale bars measure 200 μm. **B** and **C **Quantification of mineralization using the spectrophotometric Alizarin red assay. SaOS-2 cells were grown onto *N*,*O*-CMC hydrogels supplemented with different concentrations of (**B**) *S. tortuosum* ethanol extract (light blue) or aqueous extract (dark blue) or (**C**) mesembrine. The hydrogel without extract or pure compound was used as control. Biomineral formation was determined after incubation of the cells in the presence of MAC for 5 days. **C** The results were normalized for the cell number by measuring total DNA. Data are the means ± SD from (**B**) 3 independent experiments (cell cultures) with 3–4 technical replicates (*p < 0.05, ***p < 0.001; two-way ANOVA with extraction method and concentration as fixed factors; multiple comparisons with Tukey post-hoc testing and Holm correction) and (**C**) 4 experiments (non-significant, ^#^*p* > 0.05; Students *t*-test)

The effect of the *S. tortuosum* extracts in medium/FCS on mineralization was measured using SaOS-2 cells grown onto *N*,*O*-CMC hydrogels supplemented with the plant extracts. After preincubation of the cells for 3 d on Ca^2+^-hardened *N*,*O*-CMC hydrogels containing different concentrations of plant extract, osteogenic cocktail (MAC) was added to induce hydroxyapatite deposition and incubation was continued for 5 days. The results show that cells incubated in medium without MAC are only weakly stained with “OsteoImage” dye, showing a distinct green fluorescence (Fig. 5A, a and b). In contrast, in the presence of MAC, individual SaOS-2 cells, both in assays without (Fig. 5A, c and d) and with the plant extract (Fig. 5A, e and f), show a bright brilliant green staining indicating strong hydroxyapatite formation.

The “OsteoImage” assay is a sensitive assay (even more sensitive than Alizarin red S, which binds to calcium only [42]), but does not allow a quantitative assessment of mineral formation. Therefore, in parallel, the quantitative spectrophotometric Alizarin Red S assay was performed. It was found that the biomineral formation by the cells grown on the hydrogel containing the plant extracts significantly increases with increasing extract concentrations compared to the cells grown on the hydrogel without extract (Fig. 5B). A significant increase to 126.3% and 157.5% was found already at a concentration of 100 µg/mL of the plant ethanol extract and aqueous extract, respectively. Pairwise comparisons between the extraction methods showed a significantly stronger response for the aqueous extract compared to the ethanol extract (*p* < 0.05) at a concentration of 100 µg/mL, whereas no significant difference between both extracts was found at 1 mg/mL.

Mesembrine (pure compound), on the other hand, causes only a slight, but not significant (*p* > 0.05), increase in cell mineralization at a concentration of 10 µg/mL (Fig. 5C); higher concentrations were found to be cytotoxic for SaOS-2 cells (not shown).

For comparison, the effects of sertraline and fluoxetine on the mineralization of SaOS-2 cells were examined at a concentration of 10 µg/mL; this concentration is yet non-cytotoxic and corresponds to the concentration at which mesembrine exhibits weak mineralization-inducing activity (see Fig. 5C). It was found that, at the selected concentration and under the same conditions as mesembrine, both compounds exerted a slight—though not statistically significant (*p* > 0.05)—inhibitory effect on the mineralization of SaOS-2 cells compared to the control (sertraline: 0.43 ± 0.03 nmol alizarin red/µg DNA; fluoxetine: 0.41 ± 0.04 nmol alizarin red/µg DNA; control: 0.48 ± 0.04 nmol alizarin red/µg DNA; *n* = 6).

### Effect of plant extract on ALP activity of SaOS-2 cells

**Fig. 6:**
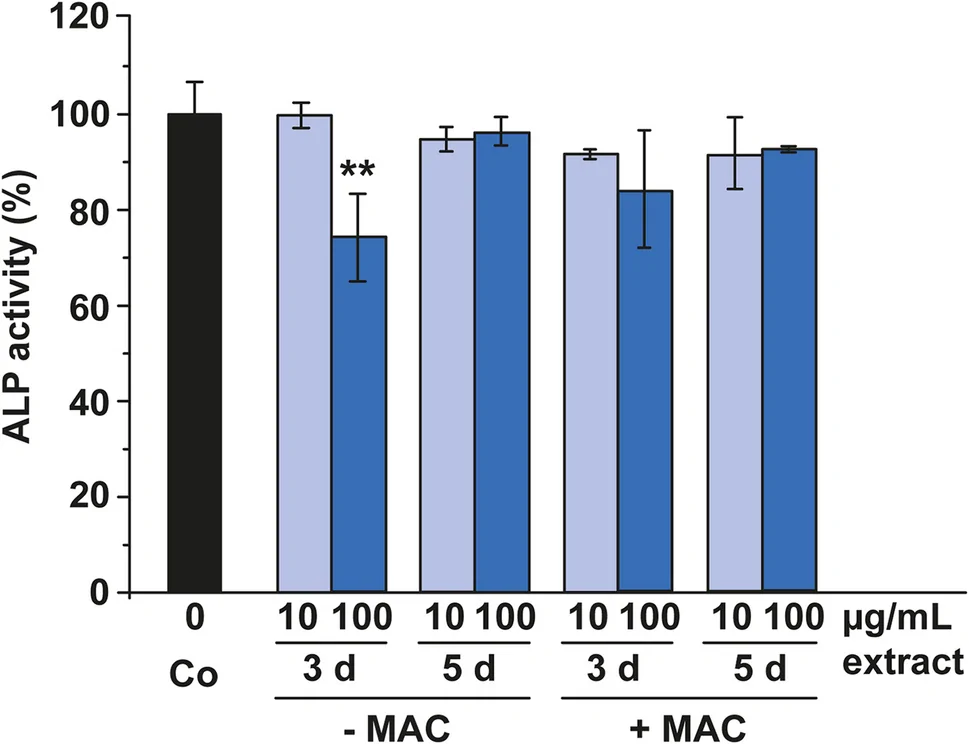
Effect of *S. tortuosum* extract on ALP activity of SaOS-2 cells. After a preincubation period of 24 h, plant ethanol extract (10–100 µg/mL) in medium/FCS was added to the cell suspension and the cells were cultivated in the absence of presence of MAC for 3 or 5 days. Thereafter, the cells were lysed and ALP activity in the supernatants was determined colorimetrically using *p*-nitrophenyl phosphate (*p*-NPP) as substrate. The results were normalized for cell number by determining the total DNA content. The values are presented as percent of control (Co; cell culture medium only) set at 100%. Means ± SD from 10 experiments each (**p < 0.01; Student’s *t*-test, compared to control)

In addition to mineralization, the effect of the *S. tortuosum* extracts on the activity of the osteoblast marker ALP was determined. Incubation of SaOS-2 cell suspension with two different concentrations of the extract in medium/FCS was performed in the absence and presence of MAC for 3 and 5 days after preincubation of the cells in the absence of these additives, as described in Methods. It was found that the *S. tortuosum* extract showed no significant effect on ALP activity, both after an incubation period of 3 and 5 days in the absence or presence of MAC (Fig. 6). Only after an incubation period of 3 days in the absence MAC, a significant decrease in ALP activity at the higher concentration tested was detected.

### Effect of the *S. tortuosum* extract on SaOS-2 cells exposed to serotonin

**Fig. 7:**
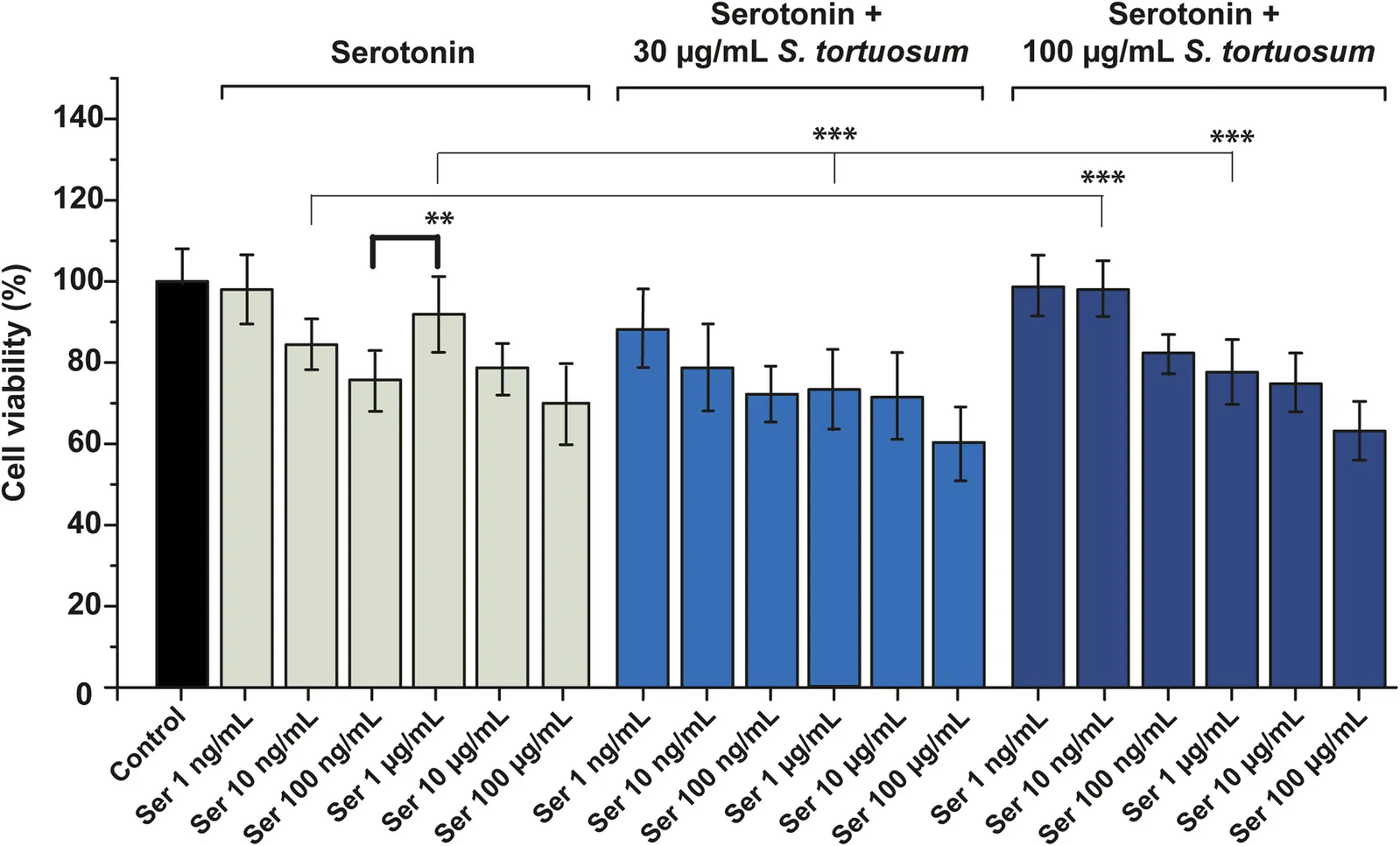
Effect of the *S. tortuosum* extract on growth/viability of SaOS-2 cells exposed to different concentrations of serotonin. The cells were incubated in the presence of increasing concentrations of serotonin, from 1 ng/mL to 100 µg/mL (final concentrations in assay), in the absence or presence of 30 µg/mL or 100 µg/mL of the ethanol extract of *S. tortuosum*. The results presented are the means ± SEM of three independent experiments with *n* = 5 each. The significances between the assays with 1 µg/mL of serotonin (transient increase of cell viability due to the activity of the 5-HT transporter) in the absence and the presence of the two extract concentrations are indicated (***p < 0.001). Only in the assays without extract, a significant difference in cell viability between the assay with 100 ng/mL and 1 µg/mL of serotonin is found (**p < 0.01; transient increase in cell viability due to the removal of extracellular serotonin by the 5-HT transporter; highlighted in Fig. 7). In addition, at a low serotonin concentration (10 ng/mL), the cell viability in the presence of 100 µg/mL of *S. tortuosum* extract significantly differed from that without extract (***p < 0.001). Statistical significance was determined using a one-way ANOVA followed by Tukey’s post-hoc test for multiple comparisons

Increasing concentrations of serotonin caused a typical effect on the growth of SaOS-2 cells, as determined by using the PrestoBlue assay. The results revealed that already at a very low concentration of 10 ng/mL of serotonin the viability of the cells started to decrease after an incubation period of 48 h, an effect, which became significant at a concentration of 100 ng/mL (Fig. 7). Thereafter, at 1 µg/mL of serotonin, the cell viability increased again, reaching 96.1% of the level in the serotonin-free control, to continue to decrease again at higher (10 µg/mL and 100 µg/mL) concentrations (Fig. 7).

In order to study the effect of the *S. tortuosum* extract on the serotonin-caused effects on growth/viability of SaOS-2, the cells were exposed to increasing concentrations of serotonin solution supplemented with 30 µg/mL or 100 µg/mL of *S. tortuosum* extract (dissolved in medium/FCS); Fig. 7. At both concentrations tested, the extract alone had no negative or positive effect on the growth/viability of the cells in PrestoBlue assay (see Fig. 2B). It was found that, despite its stimulatory effect on mineralization of SaOS-2, the extract did not enhance but significantly suppressed the transient increase in cell growth/viability at 1 µg/mL of serotonin (Fig. 7).

The statistical analysis of the results using one-way ANOVA followed by Tukey procedure revealed a significant, concentration-dependent effect on normalized cell viability (% of control) compared to the control for serotonin alone (***p = 1.4 × 10^− 5^), serotonin plus 30 µg/mL *S. tortuosum* extract (***p = 4.1 × 10^− 5^), and, even more pronounced, for serotonin plus 100 µg/mL *S. tortuosum* extract (***p = 4.6 × 10^− 7^). To compare the three treatments at matching concentrations, post-hoc pairwise comparisons (Holm adjustment for multiple testing) were performed. At 1 µg/mL serotonin, the viability of the cells was significantly higher in the absence of *S. tortuosum* extract, whereas addition of this extract significantly reduced cell viability at both tested concentrations compared to serotonin alone (both *p*-values < 0.001).

### Chemical composition of *S. tortuosum* extract

Using LC-MS it was found that the alkaloid fraction of *S. tortuosum* aqueous extract primarily consists of two major constituents, with compound 1 eluting as a broad peak between 5.5 and 6.0 min and compound 2 eluting at 5.94 min (Fig. S1 in Supplementary Material). The base peaks of the full scan mass spectra of compounds 1 and 2 appear at *m/z* 290.1762 and *m/z* 288.1605, respectively (Figs. S2 and S3). Assuming that these are the [M + H]^+^ ions of the two compounds, their molecular formulae could be calculated from the accurate masses as C_17_H_23_NO_3_ and C_17_H_21_NO_3_, respectively (Table 1). These formulae correspond to those of mesembrine and mesembrenone, two of the major compounds previously identified in *S. tortuosum* [8, 38]. Furthermore, when the MS^E^ data of compounds 1 and 2 was compared to the mass spectrometry database data [39], mesembrine and mesembrenone were some of the top matches with compounds 1 and 2. Based on this information, compounds 1 and 2 were putatively identified as mesembrine and mesembrenone, respectively.

**Table 1:** Major compounds putatively identified in the aqueous extract of *Sceletium tortuosum*

Compound	t_*R*_(min)	Compound	m/z value of [M + H]^+^	Mass error (ppm)	Chemical formula	Chemical structure
1	5.5-6.0	Mesembrine	290.1762	2.1	C_17_H_23_NO_3_	
2	5.94	Mesembrenone	288.1605	1.7	C_17_H_21_NO_3_	

The total phenolic content of the ethanol extract of *S. tortuosum* was determined to be 211.3 ± 2.4 mg GAE/g. The phenolic content of the aqueous extract was somewhat lower but still substantial (155.8 ± 4.1 mg GAE/g).

## Discussion

Serotonin has two antagonistic effects on bone formation depending on the site where it is synthesized [43]. Serotonin synthesized in the central nervous system acts as a neurotransmitter that causes increased bone formation and reduced bone resorption. Peripherally produced serotonin released in the circulation, on the other hand, leads to a reduction in bone cell maturation and mineralization [19]. Therefore, SSRIs used in therapy of depression and acting as inhibitors of the serotonin-reuptake transporter (5-HT transporter), which is also expressed by bone cells, can have adverse side-effects on bone metabolism and function. The inhibition of the 5-HT transporter by SSRIs leads to increased extracellular levels of serotonin and thus an amplification of the inhibitory effects of serotonin on osteoblast differentiation and mineralization. These adverse effects of SSRIs, leading to impaired fracture healing, could also be demonstrated in vivo, in animal experiments in mice treated with these agents such as fluoxetine [44] or sertraline [45]. In patients, osteoporosis is particularly observed after long-term treatment with these and other SSRIs, often requiring discontinuation of treatment [46].

In the present contribution we asked whether extracts of the succulent herb *S. tortuosum*, which is commercialy used for treatment/ameloriation of depressive conditions, also show these negative side-effects on bone tissue. Since *S. tortuosum* is traditionally consumed by chewing, in addition to the ethanolic extract, aqueous extracts of this plant were also investigated. We show that a 48-h exposure of the osteoblastic cell line SaOS-2 to serotonin leads to a reduction in cell growth/viability even at a concentration of 10 ng/mL – and significantly at a concentration of 100 ng/mL (Fig. 7). This serotonin-induced decline in cell growth/viability has also been demonstrated in previous studies on osteoblasts or osteoblast-like cells before [47], including SaOS-2 cells [21], and has been causally linked to the increased incidence of osteoporosis in patients treated with SSRIs. Surprisingly, we found that both extracts significantly increase the mineralization of SaOS-2 cells. This finding was particularly surprising because the *S. tortuosum* extract suppressed, at nontoxic concentrations, the transient increase in growth/viability of SaOS-2 cells occuring at a serotonin concentration of 1 µg/mL during incubation of the cells with increasing amounts of the biogenic amine (Fig. 7). Initially, at very low serotonin concentrations in the nanomolar range, an inhibition of osteoblast proliferation, differentiation, and mineralization is observed; at higher concentrations in the micromolar range, this effect is attenuated. The resulting transient increase in cell viability is characteristic of serotonin [48]; it is caused by the removal of extracellular serotonin as a result of the 5-HT transporter activity [2]. The suppression of the latter effect by the *S. tortuosum* extract can be attributed to the inhibition of the 5-HT transporter by mesembrine, the main component of *S. tortuosum* responsible for the pharmacological, anti-depressive activity of this plant [12, 15]. Therefore, it is unlikely that the mineralization stimulatory activity of the *S. tortuosum* extract is due to a reduction in extracellular serotonin levels. The opposite is expected due to the inhibition of serotonin uptake by the cells.

In further experiments, we demonstrate that *S. tortuosum* extracts exhibit a significant antioxidative activity both extra- and intracellularly. A free radical scavenging activity of *S. tortuosum* extracts in DPPH assay has already been found and described by one of us previously; the IC_50_ value of 16.7 µg/mL for the ethanol extract (Fig. 4A) was in the same concentration range as the previously reported value of 49.0 µg/mL [10]. We now show that this plant also exhibits a significant antioxidative activity in assays using the cell-permeant probe H_2_DCF-DA, which allows the detection of ROS generated intracellularly.

Using LC-MS/MS it was determined that the alkaloid fraction of the aqueous extract of *S. tortuosum* primarily consists of two major compounds, namely mesembrine and mesembrenone. *S. tortuosum* is known to contain a series of bioactive mesembrine-type alkaloids, including mesembrine, mesembrenone and mesembrenol, with the principal active component being mesembrine [8]. These compounds are also known to take part in oxidation and reduction reactions during fermentation [38]. In a recent study [49], a larval zebrafish model was used to show that pure mesembrine has a higher anxiolytic effect compared to its analogues. On the other hand, when a mixture of mesembrine and some of its analogues was tested, even higher effects could be achieved compared to the pure mesembrine alone.

Determination of mineralization-inducing activity showed that, in contrast to the *S. tortuosum* extract, mesembrine, at a concentration of 10 µg/ml, caused, if at all, only a slight, not very significant, increase in mineralization in SaOS-2 cells compared to the control. Higher concentrations of the compound were found to be cytotoxic on SaOS-2 cells.

In a further series of experiments, the effects of the two SSRIs sertraline and fluoxetine on the mineralization of SaOS-2 cells were also investigated. Both drugs belong to the most frequently prescribed SSRIs. Their impact on bone formation has already been demonstrated in both in vitro and animal studies; e.g., it has been shown that sertraline inhibits bone healing in a mouse calvarial defect model [45] and fluoxetine impairs mineralization in a mouse model of femoral fractures [44]. Our results showed that, at the chosen concentration (10 µg/mL, the concentration at which mesembrine produced a slight but non-significant effect; *p* > 0.05), both drugs did not promote the mineralization of SaOS-2 cells but rather inhibited it. A similar inhibitory effect has been described for citalopram; in the same cell system (SaOS-2), citalopram reduced mineralization to approximately 85% of the control level at a concentration of 2 µg/mL [50].

The result with mesembrine is in line with previous studies showing that this compound is only a very weak inhibitor of phosphodiesterase-4 (PDE4), which increases cellular cAMP levels, while this alkaloid acts as a potent blocker of the 5-HT transporter (*K*_i_ 1.4 nM [12]). Based on these results and considering the low alkaloid content of the *S. tortuosum* extract (≤ 1% [wt/wt]; see also [17]), we conclude that a possible stimulatory effect of the extract on mineralization *via* inhibition of PDE4 by mesembrine can be neglected, although it cannot be excluded that this effect is partly caused by mesembrenone, the second most prevalent alkaloid in the extract, which inhibits this enzyme more strongly than mesembrine [12]. However, as demonstrated in this study, exposure of SaOS-2 to the *S. tortuosum* extract did not lead to increased ALP activity (a marker of osteogenic differentiation) but rather inhibited the enzyme at the highest concentration tested (Fig. 6). Therefore, it seems unlikely that cAMP signaling plays a crucial role in the induction of mineralization by the *S. tortuosum* extract.

The reason why the *S. tortuosum* extract increases mineralization but exerts no stimulatory effect on ALP activity remains unclear. This could be due to the presence of inhibitors of this enzyme, as observed with certain plant extracts—particularly those rich in polyphenols (e.g., quercetin-7-*O*-galactoside in *Hibiscus rosa-sinensis* extracts [51]). Furthermore, secondary metabolites within plant extracts acting as metal chelators could influence ALP activity, given that this enzyme is a metalloenzyme dependent on Zn^2+^ and Mg^2+^ ions. The observation that the *S. tortuosum* extract exhibits an inhibitory effect on ALP at the highest concentration tested might suggest the presence of such an inhibitor (Fig. 6). On the other hand, SaOS-2 cells are characterized by an exceptionally a high baseline ALP activity, even in the absence of agents that increase cellular cAMP level [52, 53], sufficient to support the mineralization process. ALP is an ectoenzyme that functions at the cell surface, where it cleaves extracellular substrate molecules such as β-glycerophosphate contained in the mineralization activation cocktail (MAC) used in our assay. Increased mineralization could therefore also result from the translocation of ALP to the cell surface, as we have previously demonstrated [54]. This would lead to enhanced mineralization without altering the total ALP activity measured in cell lysates, as determined in our study.

*S. tortuosum* extracts are characterized by a high content of compounds with antioxidative activity; in an earlier study, we reported a phenolic content of 171.89 ± 0.05 mg GAE/g for the ethanol extract [10]. Here we found that, similar to the ethanol extract (211.3 ± 2.4 mg GAE/g), the aqueous extract of *S. tortuosum* also has a high phenol content (155.8 ± 4.1 mg GAE/g). This explains the strong radical scavenging activity of the extracts demonstrated in our study. Mesembrine itself showed no radical scavenging activity in the DPPH test.

Therefore, we assume that the mineralization-promoting effect of *S. tortuosum* extracts is primarily due to the antioxidant, radical scavenging activity of the phenolic constituents of the extracts, which could enhance the activity of the alkaloid constituents. It is known that ROS are crucially involved in bone formation and remodeling *via* different, partly opposing mechanisms, including both cell differentiation- and apoptosis-inducing pathways [55]. Oxidative stress based on an inballance of ROS generating and inactivating systems is also causatively involved in the pathogenesis of osteoporosis by increasing osteoclastogenesis and reducing osteogenesis and mineralization [26].

**Fig. 8:**
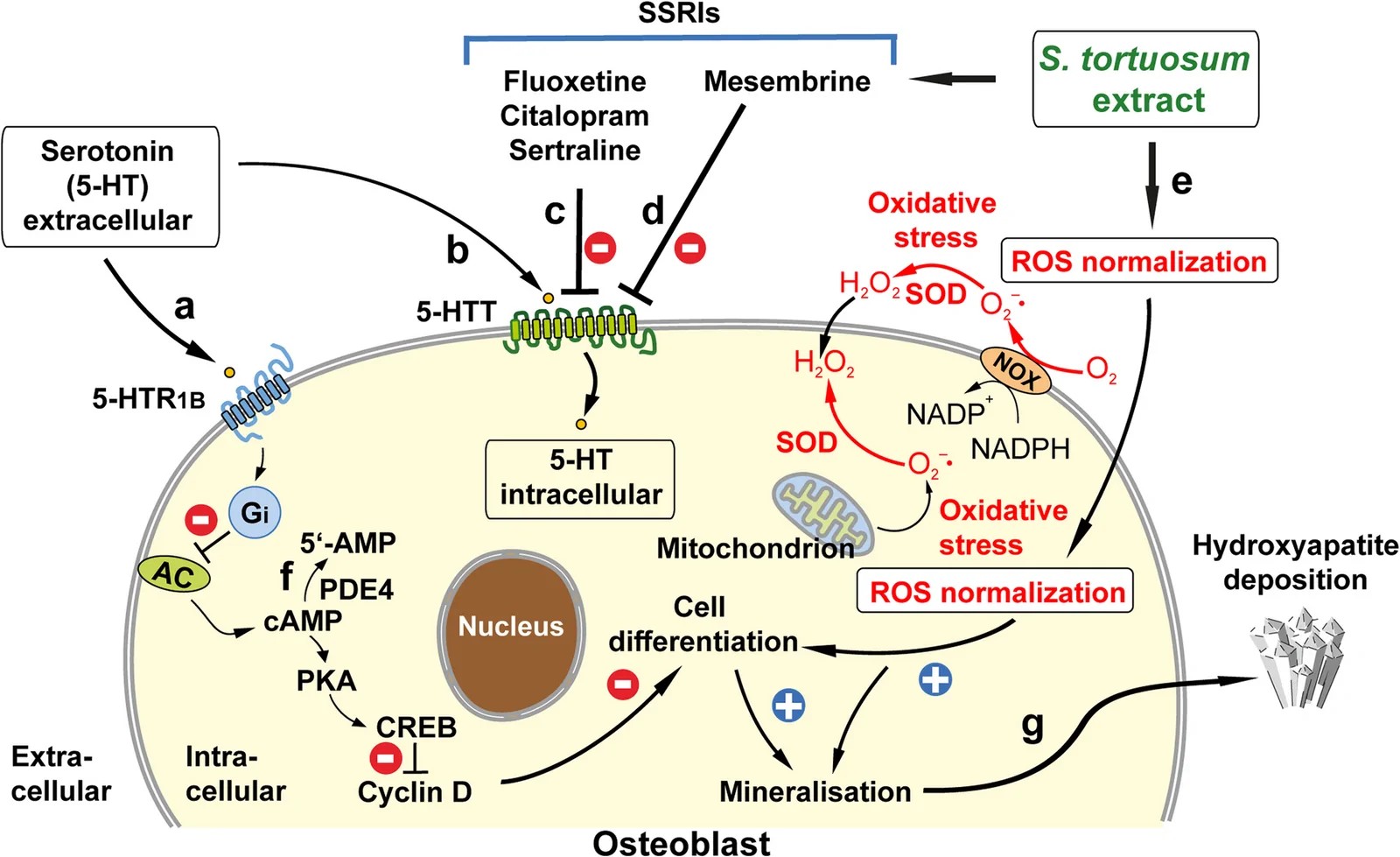
Proposed mechanism of action of *S. tortuosum* extract on osteoblasts. In the absence of extract, serotonin binds to the 5-HT_1B_ receptor (5-HTR_1B_) (**a**), leading to impaired osteoblast differentiation and function (hydroxyapatite formation) *via* inhibition of adenylate cyclase (AC) and reduced phosphorylation of the transcription factor CREB by cAMP-dependent protein kinase A (PKA). This effect is transiently reversed with increasing concentrations of serotonin through cellular uptake of the biogenic amine *via* the serotonin reuptake transporter (5-HT transporter; 5-HTT) (**b**), resulting in a reduction of the extracellular serotonin level, an effect that is suppressed by SSRIs (**c**) used as antidepressants, e.g., fluoxetine, citalopram and sertralin, but also mesembrine (**d**) the psychoactive constituent of *S. tortuosum*. However, based on the results presented, the opposite effect, not a reduced but an increased mineralization, is observed after exposure of osteoblasts to the *S. tortuosum* extract. The latter effect is explained by the antioxidative activity of the extract, both extra- and intracellularly (**e**), in addition to an inhibition of phosphodiesterase-4 (PDE4) (**f**). Through neutralization of ROS, which at higher concentrations impair mineralization, the plant extract helps to reverse the adverse effects of SSRIs on the function of osteoblasts and restore their mineralization ability (**g**)

Although this is still very preliminary given the current state of knowledge, Fig. 8 shows a schematic representation of the presumed mode of action of *S. tortuosum* extracts on osteoblasts, leading to inhibition of the serotonin reuptake transporter while simultaneously resulting in enhanced mineralization of the cells. Binding of serotonin to the serotonin receptor on the osteoblast causes inhibition of protein kinase A (PKA)-mediated phosphorylation of the transcription factor CREB (cAMP response element binding protein), resulting in reduced proliferation and mineralization of the cells [48, 56]. In the absence of *S. tortuosum* extract, this effect, which is already observed at serotonin concentrations in the nanomolar range, is mitigated at increasing concentrations of the biogenic amine, characteristically in the micromolar range (at around 1 µg/mL), an effect that is abolished in the presence of the extract (see Fig. 7) or pure compound, mesembrine [12, 15]. However, most likely due to its high content of antioxidants, the inhibition of serotonin reuptake is not associated with a reduction of mineral deposition by the osteoblast cells, but with an enhanced mineralization process. It is assumed that the *S. tortuosum* causes a reduction (normalization) of ROS levels both extra- and intracellularly, thereby increasing mineralization and reversing the adverse effect of the inhibition of the serotonin reuptake transporter on osteoblast function.

Based on the finding that serotonin inhibits the proliferation of SaOS-2 cells, we conclude that serotonin primarly acts on this human osteoblast cell line *via* binding to the 5-HT_1B_ receptor, in line with previous results [21], although a binding to 5-HT_2A_, maybe at higher concentrations, cannot be excluded due to the inhibition of serotonin reuptake with increasing concentrations of serotonin, as shown in the present contribution, an effect that was not abolished in the presence of the plant extract. It has been reported that the reuptake of serotonin by osteoblasts becomes downregulated after phosphorylation of the 5-HT transporter [57].

The results presented indicate that *S. tortuosum* and extracts from this indigenous medicinal plant are not only of interest for the treatment of psychiatric disorders but may also possess the potential to serve as dietary supplements for the prophylaxis or supportive treatment of osteoporosis. However, it should be noted that the mineralization-promoting effect of the plant extracts in the present study was demonstrated using osteosarcoma-derived SaOS-2 cells. Further investigations involving primary osteoblasts and osteoclasts, as well as animal experiments and human studies, will be required. *S. tortuosum* extracts have previously been shown to also possess antiviral activity by inhibiting HIV-1 reverse transcriptase [10]. This latter finding is particularly significant for countries where *S. tortuosum* is traditionally consumed by local population, given that sub-Saharan Africa is the global region with the highest prevalence of HIV/AIDS [58]. Even more, HIV infections have been shown to be associated with an increased incidence of osteoporosis [59]. This virus can impair the tuned interaction between bone forming osteoblasts and bone degrading osteoclasts by decreasing the expression and secretion of osteoprotegerin by osteoblasts, resulting in an increased osteoclast activity [60]. Future studies need to clarify whether extracts of *S. tortuosum*—in addition to their antidepressant and anxiolytic effects, which were recently convincingly demonstrated in Wistar rats [61]—also improve bone health in vivo in animal models. A critical review was recently published addressing current preclinical and clinical studies on *S. tortuosum* and its alkaloids, as well as the need to further substantiate their promising, medically relevant effects [62]. In this context, it should also be noted that the observed biological effects may depend significantly on the chemotype of *S. tortuosum*—as demonstrated by the comparison of wild-harvested plants from different geographical origins—including effects on neurotransmitter concentrations (such as serotonin) in various regions of the central nervous system [63].

## Conclusions

This study demonstrates that *S. tortuosum* extracts have a significant mineralization-promoting effect on bone-forming SaOS-2 cells. This property contrasts with SSRIs commonly used in antidepressant therapy that impair bone mineral formation as a side effect during long-term treatment. Surprisingly, this induction of mineralization was observed even though the *S. tortuosum* extract, like other antidepressants, inhibits the transient mitigating effect on serotonin-induced suppression of SaOS-2 viability upon increasing concentrations of the biogenic amine. The ALP activity was not changed. Furthermore, the *S. tortuosum* extracts were found to exhibit a potent ROS scavenging activity not only in the DPPH assay but also in cells, in SaOS-2, as measured with the cell-permeant H_2_DCF-DA probe. Furthermore, LC-MS analysis revealed that the major alkaloid components of the aqueous extract of *S. tortuosum* are mesembrine and mesembrenone, which are known to be involved in oxidation and reduction reactions during fermentation. Based on the presented results, it is suggested that balancing ROS levels is critically involved in the mechanism underlying the stimulatory in vitro effect of *S. tortuosum* extracts on bone mineral formation demonstrated in this study. However, it cannot be ruled out that other mechanisms are also involved, such as the modulation of the cAMP signaling pathway through a potential effect of the extracts on serotonin reuptake and PDE4 activity. It is concluded that *S. tortuosum* and its extracts could be of interest as dietary supplements for individuals at risk of osteoporosis due to their mineralization-inducing activity—particularly in the context of adjuvant therapy for patients with depression disorders, as they may counteract the adverse effects of SSRIs (one of the most frequently prescribed classes of drugs in pharmacotherapy) on bone tissue. However, further studies are required before this conclusion can be definitively drawn.

## Supplementary Information

Supplementary Material 1.

## Data Availability

The data supporting the results are included in this paper and the Supplementary Information and are available from the corresponding authors upon reasonable request.
