# Supplementary material for: Mitigation of serotonin-caused suppression of osteoblast function by extracts of the psychoactive indigenous plant Sceletium tortuosum
Source: BMC Complement Med Ther. 2026 Aug 22;26:255. doi: 10.1186/s12906-026-05540-x (PMC13499329; doi:10.1186/s12906-026-05540-x)
Supplement: Supplementary file 1 [file 12906_2026_5540_MOESM1_ESM.docx]

**Supplementary Material**

**Mitigation of serotonin-caused suppression of osteoblast function by extracts of the psychoactive indigenous plant** ***Sceletium tortuosum***

Heinz C. Schröder^1,2,^*, Matthias Wiens^1^, Shunfeng Wang^1^, Meik Neufurth^1^, Stefan Louw^3^, Oluwagbenga Johnson^4,5^, Susanne Hoff^6^, Xiaohong Wang^1^, Werner E.G. Müller^1,^*, Petrina Kapewangolo^7^ and Fransina Shafombabi^7^

^1^ ERC Advanced Investigator Group, Institute for Physiological Chemistry, University Medical Center of the Johannes Gutenberg University, Duesbergweg 6, 55128 Mainz, Germany e-mail: hschroed@uni-mainz.de; wiens@uni-mainz.de; wsfwmkx@gmail.com; mneufurt@uni-mainz.de; wang013@uni-mainz.de; wmueller@uni-mainz.de

^2^ NanotecMARIN GmbH, Mühlstr. 19, D-55218 Ingelheim am Rhein, Germany; e-mail: schroeder@nanotecmarin.de

^3^ Department of Physics, Chemistry and Material Science, University of Namibia, Private Bag 13301, 340 Mandume Ndemufayo Ave, Pionierspark, Windhoek, Namibia, e-mail: slouw@unam.na

^4^ Department of Mining and Metallurgical Engineering, University of Namibia, Eng. José Eduardo dos Santos Campus, P.O. Box 3624, Ongwediva, Namibia; e-mail: ojohnson@unam.na

^5^ Department of Chemical Engineering, University of South Africa, Pretoria, South Africa; e-mail: johnson.gbenga@gmail.com

^6^ Mediplants Namibia Cc, P.O. Box 2017, Windhoek, Namibia; e-mail: susanne@mediplantsnamibia.com

^7^ Department of Chemistry and Biochemistry, University of Namibia, Windhoek, Namibia; e-mail: pkapewangolo@unam.na

**Fig. S1.** **(A)** Electrospray positive ionisation total ion chromatogram, **(B)** the extracted ion chromatogram of *m/z* 290, the [M+H]^+^ ion of compound **1** and **(C)** the extracted ion chromatogram of *m/z* 288, the [M+H]^+^ ion of compound **2_,_** of the LC-MS analysis of the aqueous extract of *S. tortuosum*.

**Fig. S2.** Full scan **(A)** and MS^E^ **(B)** spectra of compound **1**, putatively identified as mesembrine in the aqueous extract of *S. tortuosum*.

**Fig. S3.** Full scan **(A)** and MS^E^ **(B)** spectra of compound **2**, putatively identified as mesembrenone in the aqueous extract of *S. tortuosum*. (Using an extracted ion chromatogram approach, it was determined that the peaks observed at *ca*. *m/z* 204, 219, 276 and 290 of the MS^E^ spectrum (B) are present due to co-elution of compounds **2** with compound **1**.)
